# Lymphatic Defects in Zebrafish *sox18* Mutants Are Exacerbated by Perturbed VEGFC Signaling, While Masked by Elevated *sox7* Expression

**DOI:** 10.3390/cells12182309

**Published:** 2023-09-19

**Authors:** Silvia Moleri, Sara Mercurio, Alex Pezzotta, Donatella D’Angelo, Alessia Brix, Alice Plebani, Giulia Lini, Marialaura Di Fuorti, Monica Beltrame

**Affiliations:** 1 Dipartimento di Bioscienze, Università degli Studi di Milano, Via Celoria 26, 20133 Milan, Italy; 2 Dipartimento di Biotecnologie e Bioscienze, Università degli Studi di Milano-Bicocca, Piazza della Scienza 2, 20126 Milan, Italy

**Keywords:** Sox18, SoxF transcription factors, VEGFC, lymphatic development, zebrafish

## Abstract

Mutations in the transcription factor-coding gene *SOX18*, the growth factor-coding gene *VEGFC* and its receptor-coding gene *VEGFR3/FLT4* cause primary lymphedema in humans. In mammals, SOX18, together with COUP-TFII/NR2F2, activates the expression of *Prox1*, a master regulator in lymphatic identity and development. Knockdown studies have also suggested an involvement of Sox18, Coup-tfII/Nr2f2, and Prox1 in zebrafish lymphatic development. Mutants in the corresponding genes initially failed to recapitulate the lymphatic defects observed in morphants. In this paper, we describe a novel zebrafish *sox18* mutant allele, *sa12315*, which behaves as a null. The formation of the lymphatic thoracic duct is affected in *sox18* homozygous mutants, but defects are milder in both zygotic and maternal-zygotic *sox18* mutants than in *sox18* morphants. Remarkably, in *sox18* mutants, the expression of the closely related *sox7* gene is elevated where lymphatic precursors arise. Sox7 could thus mask the absence of a functional Sox18 protein and account for the mild lymphatic phenotype in *sox18* mutants, as shown in mice. Partial knockdown of *vegfc* exacerbates lymphatic defects in *sox18* mutants, making them visible in heterozygotes. Our data thus reinforce the genetic interaction between Sox18 and Vegfc in lymphatic development, previously suggested by knockdown studies, and highlight the ability of Sox7 to compensate for Sox18 lymphatic dysfunction.

## 1. Introduction

SOX18, and the closely related SOX7 and SOX17 proteins, belong to the SOXF group of Sry-related HMG-box transcription factors [1], which play various roles in cardiovascular and lymphatic development [2]. Mutations in *SOX18* underlie dominant and recessive forms of hypotrichosis–lymphedema–telangiectasia syndrome (HLTS) [3], combining defects in hair/eyelashes/eyebrows with lymphatic dysfunction, cutaneous red stains and, in some cases, renal failure (HLTRS) [4]. Recessive mutations correspond to missense mutations causing substitutions in key amino acids of the HMG-box DNA binding domain, while de novo dominant mutations correspond to nonsense mutations or frameshifts, leading to premature termination codons downstream of the DNA-binding domain coding sequence. The reported *SOX18* pathogenic variants are very few, in a dozen of families, and yet the phenotypic spectrum is quite varied in the reported patients. Of note, two patients did not present with lymphatic dysfunction [5,6], whereas others already presented with lymphedema at birth and one died in utero with non-immune hydrops fetalis ([3,7] and references therein).

The spontaneous *ragged* mutants, named in the 1950s and 1960s for their characteristic sparse fur, represent the murine counterpart of the disease, with different alleles of increasing severity all associated with single-base deletions generating truncated, dominant-negative SOX18 proteins lacking transactivation ability [8,9]. The most severe allele, *Sox18-RaOp*, was found to cause edema, chylous ascites and superficial hemorrhage in neonates in the heterozygous state, while being embryonic lethal in the homozygous state with signs of severe lymphatic and vascular dysfunction [10,11]. *Sox18*-null mutant mice, produced by gene targeting, were viable and displayed only mild coat defects in the originally described mixed genetic background [12], raising the hypothesis of functional redundancy with the other SoxF group genes. In a C57BL/6 pure background, *Sox18^−/−^* mice were instead embryonic lethal by 14.5 days post-coitum (dpc) and showed gross subcutaneous edema like *Sox18-RaOp* homozygous embryos [11].

SOX18 is expressed in the endothelial cells (ECs) of all forming vessels in mice, including a subset of cells of the cardinal vein (CV), which also express the transcription factor NR2F2/COUP-TFII, prior to the expression of PROX1, a key transcription factor in lymphatic development [11,13,14]. SOX18 and COUP-TFII are able to directly activate the expression of *Prox1*, thus turning blood ECs (BECs) into lymphatic EC (LEC) progenitors. The other SOXF transcription factors, SOX7 and SOX17, were shown to share the ability to activate *Prox1* transcription in vitro. However, they are not normally expressed in the CV during lymphatic development. When *Sox18* is mutated, their expression is upregulated in the CV in the mixed genetic background and not in the C57BL/6 pure background, thus explaining the dramatic difference in lymphatic phenotype [15].

Vascular Endothelial Growth Factor C (VEGFC)/Vascular Endothelial Growth Factor Receptor 3 (VEGFR3) signaling plays a crucial role in lymphatic development (reviewed in [16]). In particular, the egress of LEC precursors from the CV and their subsequent migration are strictly dependent on a VEGFC gradient and on VEGFR3 in mice. Mutations in *VEGFR3/FLT4*, *VEGFC*, and genes involved in the processing of VEGFC are associated with lymphatic anomalies in humans (e.g., Milroy’s disease, Congenital Primary Lymphedema of Gordon, Hennekam syndrome; reviewed in [17]).

In zebrafish, the onset of lymphatic development can be visualized with transgenic reporter lines from around 1.5 days post-fertilization (dpf), when venous and lymphatic sprouts emerge from the posterior CV (PCV) [18,19,20]. Lymphatic precursors migrate dorsally to the horizontal myoseptum, where they constitute a transient population of parachordal lymphangioblasts (PLs), later migrating ventrally and dorsally along arterial intersegmental vessels (aISVs) to give rise to the thoracic duct (TD), the intersegmental lymphatic vessels, and the dorsal longitudinal lymphatic vessels ([21]; reviewed in [22]). The TD forms in the trunk just below the dorsal aorta (DA), between 3 and 5 dpf, from separate segments migrating rostrally and caudally to then interconnect in a single vessel [18,23].

Initial characterization of early phases of zebrafish lymphatic development, through knockdown studies, revealed a high degree of conservation of key molecular players and signaling pathways between fish and mammals. In particular, *vegfc* morphants showed an absence of TD formation [18,23], as *vegfc* is required for venous and lymphatic sprouting from the PCV [20]. The key relevance of Vegfc-Vegfr3 signaling for lymphatic development in zebrafish was later confirmed by the characterization of mutants in *vegfr3*/*flt4* and *vegfc* identified through forward genetic screens [24,25,26].

Knockdown studies have also suggested conserved roles in lymphatic development for zebrafish homologues of *Prox1*, *Coup-TFII*, and *Sox18* [18,27,28]. As for *sox18*, our and other groups have shown that the *soxF* genes *sox7* and *sox18* are largely coexpressed in the forming axial vessels and intersegmental vessels during vasculogenesis and primary angiogenesis, and that they play redundant roles in arteriovenous differentiation of ECs [29,30,31]. We noticed that *sox18* expression persists in the PCV, while *sox7* expression fades away in the PCV at developmental stages prior to secondary venous and lymphatic sprouting [28]. Knockdown of *sox18* with morpholinos, either interfering with splicing or blocking translation, affects early trunk lymphatic development: lymphatic precursors at the horizontal myoseptum are reduced and TD formation is defective. Moreover, the synergistic effects of the simultaneous partial knockdown of *sox18* and *vegfc* brought us to hypothesize a genetic interaction between the two factors in zebrafish lymphatic development [28].

The initial characterization of reverse genetic mutants in *prox1a*, *prox1b*, *coup-TFII*/*nr2f2*, and *sox18* revealed no or marginal defects in TD formation, leading to the conclusion that the Prox1-Sox18-Coup-TFII transcription factor axis is dispensable for lymphatic development is zebrafish [32,33]. However, further analysis of a maternal-zygotic *prox1a* mutant pointed to a conserved crucial function of Prox1 in early phases of lymphatic differentiation [34]. Moreover, Vegfc was shown to be a key factor in enhancing *prox1a* expression in a subset of cells of the PCV, which later give rise to lymphatic precursors migrating towards the horizontal myoseptum [34].

This prompted us to reevaluate the role of Sox18 in zebrafish lymphatic development through the characterization of a novel mutant allele, *sox18^sa12315^*, from the Zebrafish Mutation Project [35]. Here, we combined this new mutant allele with partial *vegfc* knockdown to show the role of Sox18 in driving lymphatic patterning, and we unveiled the ability of Sox7 to compensate for Sox18 loss.

## 2. Materials and Methods

### 2.1. Zebrafish Lines and Maintenance

Zebrafish were raised and maintained according to established techniques [36] and to the European recommendations [37] and Italian regulations. The following lines were used: *sox18^sa1231^* (from the Wellcome Sanger Institute, through the European Zebrafish Resource Center; [35]), *Tg(fli1:EGFP)^y1^* [18,38], *Tg(lyve1b:DsRed)^nz101^* [39], *Tg(mrc1a:EGFP)^y251^*;*Tg(kdrl:mCherry)^y171^* [40].

### 2.2. Genotyping

The *sox18^sa12315^* zebrafish mutant line is characterized by a G/A transition, in the sequence encoding the HMG-box domain of the *sox18* gene, which causes the disruption of a restriction site for the BstNI/MvaI enzymes. Genomic DNA (gDNA) extraction was performed using incubation in lysis buffer (10 mM Tris-HCl pH 8, 1 mM EDTA pH 8, 0.3% Tween20, 0.3% glycerol) for 10 min at 98 °C, followed by 4 h of overnight digestion at 55 °C with Proteinase K (PK, 1 µg/µL final concentration) and PK inactivation for 10 min at 98 °C. gDNA was used for PCR amplification with the following primers: *sox18*-BstNI-F1: 5′-GATTGCATTTAGATGATGTTGTCCTG-3′ and *sox18-*BstNI-R1: 5′-CATCTTCTTGGGTTGTTTCTTCCTC-3′. In case of low yields, a second PCR was performed with the following internal primers: *sox18-*BstNI-F2: 5′-CAGTGCTCTGGCACTAGATTG-3′ and *sox18-*BstNI-R2: 5′-AAGCCTTGGAGAAGGAGACC-3′. PCR products were digested with MvaI, or its isoschizomer, BstNI, and analyzed on 3% agarose gels to discriminate the different genotypes, based on their digestion patterns.

### 2.3. MO Microinjections

Antisense morpholinos (MOs; Gene Tools, Philomath, OR, USA) were injected as described elsewhere [29]. As controls, we used uninjected embryos or injected a standard control oligo (std-MO), with no target in zebrafish embryos, to assess unspecific effects. We injected 0.125 or 0.25 pmol/embryo of *sox7*-MO1 [29], 0.045 pmol/embryo of *vegfc*-MO and 1 pmol/embryo of *sox18*-MO2 [28]. The sequences (5′-3′) of the morpholinos are as follows: std-MO: CCTCTTACCTCAGTTACAATTTATA; *sox7*-MO1: ACGCACTTATCAGAGCCGCCATGTG; *vegfc*-MO: AGACAGAAAATCCAAATAAGTGCAT; and *sox18*-MO2: gtgagtgtcttacCCAGCATTTTAC (intron-targeting sequence in lowercase).

### 2.4. In Situ Hybridizations

Whole-mount in situ hybridizations (ISHs) were carried out essentially as previously described [28,29,41]. The following primers were used in PCR reactions to generate DNA templates for RNA probes: *sox18,* fw: 5′-GGAGCCAGGAGTTACAAAACAC-3′, and rev: 5′-CTAATACGACTCACTATAGGGCTCCATATGTGCACCAGACTTC-3; *sox7,* fw: 5′-CCCGCTTGATAAAGATGACG-3′, and rev: 5′-CTAATACGACTCACTATAGGGTTGGAAGAGACCAGCCTCAC-3′; sox17, fw: 5′- ACGAAACAAGCGATTGGAGC-3′, and rev: 5′- CTAATACGACTCACTATAGGGTGCCATTTAAGCTGCTGACA-3′; *plvapb*/*vsg1,* fw: 5′-CTACCCACAAGTGTGACAGTGC-3′, and rev: 5′- CTAATACGACTCACTATAGGGGATCAGATTCCTTCTCCACACC-3′. Additionally, antisense DIG-labeled RNA probes for *sox7* and *cdh5* were transcribed in vitro using linearized plasmids as DNA templates [29,42].

Images were taken with a Leica MZFLIII epifluorescence stereomicroscope equipped with a DFC 480-R2 digital camera and LAS imaging software version 4.13.0 (Leica, Wetzlar, Germany).

The PCV/DA ratio in *sox18* and *sox7* ISHs was evaluated using ImageJ software version 1.47v, on images converted to 8-bit images. We set the threshold color with constant parameters and measured the number of positive pixels in 8 rectangular ROIs (regions of interest) selected in the axial vessels of the trunk. In each image, we set the ROIs in the DA (dorsal aorta) and PCV (posterior cardinal vein) under intersomitic vessels 1, 4, 7, and 10 (counting from the anus). We then calculated the ratio between the sum of all values obtained in PCV ROIs and the sum of all values of DA ROIs. Data were plotted using GraphPad Prism version 9.0.0.

### 2.5. Phenotypic Analyses

We evaluated embryo circulation in the trunk/tail region as reported in [29]. All larvae analyzed to study lymphatic development were circulating, and we scored them for TD formation essentially as described in [28], along 10 consecutive trunk segments, counted rostrally from the anus, in larvae at 5 dpf. Confocal microscopy was performed on a Nikon Eclipse-Ti inverted microscope (Nikon, Tokyo, Japan), and images were processed using Adobe Photoshop version CS6 or NIS-Viewer version 4.11.0.

### 2.6. Statistical Analyses

Statistical analyses were performed with a *t*-test or one-way ANOVA followed byDunnett’s Multiple Comparison post-test, when needed, using GraphPad Prism version 9.0.0 (GraphPad, San Diego, CA, USA). In the graphs, * and ** mark statistically significant data with a *p*-value < 0.05 and <0.01, respectively. Statistically highly significant data, with a *p*-value < 0.001 and <0.0001, are marked by *** and ****, respectively.

## 3. Results

### 3.1. The sa12315 Mutation Is a Loss-of-Function Allele of sox18

**Figure 1:**
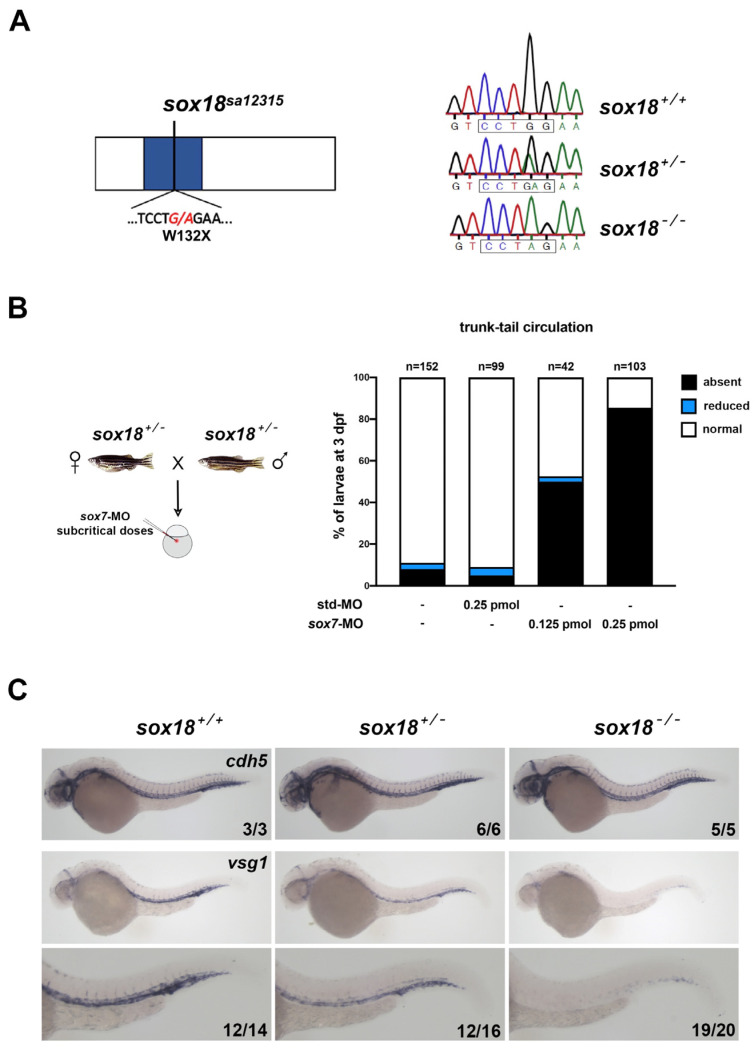
The *sox18^sa12315^* mutant behaves as expected for a null allele. (**A**) On the left is a schematic representation of the Sox18 protein, with the HMG-box domain in blue. The G > A transition and the premature stop codon introduced in the *sa12315* mutant are indicated. Fragments of the electropherograms derived using Sanger sequencing of the region surrounding the mutation in wt (*sox18^+/+^*), heterozygous (*sox18^+/−^*) or homozygous mutants (*sox18^−/−^*) are reported on the right. The restriction site for the BstNI/MvaI enzymes (boxed sequence) is disrupted by the mutation. (**B**) Embryos derived from *sa12315* heterozygote matings and injected with subcritical doses of *sox7*-MO were collected in several independent experiments and analyzed in vivo at 2 dpf and 3 dpf or fixed at around 30 hpf for ISH, as shown in (**C**). The histogram on the right shows the trunk–tail circulatory phenotypes observed at 3 dpf. In control embryos, i.e., uninjected or injected with a standard control MO (first and second bars, respectively), trunk–tail circulatory defects are present in a small percentage of embryos. On the contrary, the partial knockdown of *sox7* causes a blockage in trunk–tail circulation in a dose-dependent manner (third and fourth bars). Circulatory defects are genotype-dependent (see Table S1). (**C**) ISHs were performed on embryos derived from *sa12315* heterozygote matings and injected with subcritical doses of *sox7*-MO, as shown in (**B**), fixed at around 30 hpf. Upper panels show control ISH performed with the endothelial marker *cdh5,* showing no gross alteration in embryos of the three different genotypes. Lower panels show ISHs performed with a probe for *vsg1/plvapb*, whose expression was particularly downregulated in double partial *sox7/sox18* morphants [29]. Higher magnification images of the trunk–tail regions of the embryos are also shown. Experiments were repeated twice; all *plavpb* stained embryos and a subset of *cdh5* stained embryos were genotyped; numbers in each image refer to a single experiment. Lateral views, anterior to the left. Pictures were taken at 40× and 63× magnification, for lower and higher magnification images respectively.

To better address the role of Sox18 in zebrafish lymphatic development, we decided to characterize the *sa12315* mutation in the *sox18* locus, generated at the Wellcome Sanger Institute within the Zebrafish Mutation Project [35]. This mutant allele corresponds to a G > A transition in the second of the two exons of the *sox18* gene, introducing a premature termination codon (W132X) within the sequence coding for the DNA-binding domain of the Sox18 transcription factor (Figure 1A). This mutation is expected to truncate the protein in the second of the three alpha helices of the HMG-box domain, thus eliminating the transactivation domain, which is more C-terminal in the wild-type form, but also disrupting the DNA-binding ability of Sox18.

To confirm that the *sa12315* mutant allele corresponds to a loss-of-function allele, we exploited the known functional redundancy between Sox18 and Sox7 in vascular development [29]. The progeny of fish heterozygous for the *sa12315* mutation were injected with subcritical doses of a *sox7* morpholino, which was already shown to cause little effects on its own but dramatic alterations in trunk–tail blood circulation when combined with the partial knockdown of *sox18* [29]. At 3 days post-fertilization (3 dpf), half of the progeny showed a circulatory phenotype in the trunk–tail region when injected with as little as 0.125 pmoles of *sox7*-MO (n = 42, 21 noncirculating embryos). Doubling the dose of *sox7*-MO (0.25 pmoles) caused over three-quarters of the injected embryos to show a circulatory phenotype (n = 103, 88 noncirculating embryos). Circulatory defects were far less pronounced in controls, i.e., uninjected or injected with a standard control MO (only around 10%, n = 152 and 99, respectively) (Figure 1B). These data strongly suggest that circulatory defects are associated with the partial knockdown of *sox7* combined with a reduction in or loss of functional Sox18 in heterozygous or homozygous *sa12315* mutants, respectively (see also Table S1, showing genotype–phenotype analysis of a small clutch of embryos).

Moreover, we characterized, at the molecular level, the effect of partial knockdown of *sox7* in the progeny of fish heterozygous for the *sa12315* mutation. We previously highlighted that *plvapb* (also known as *vsg1*) is one of the genes downregulated in *sox7* and *sox18* double partial morphants [29]. In situ hybridizations (ISHs) revealed that the endothelial *plvapb* signal is reduced in a genotype-dependent way in embryos carrying one or two *sa12315* alleles injected with a subcritical amount of *sox7*-MO (Figure 1C), whereas the expression of the endothelial marker *cdh5* is largely unaffected.

### 3.2. sa12315 Mutants Show Mild Lymphatic Defects, Which Are Exacerbated by Perturbed VEGFC Signaling

**Figure 2:**
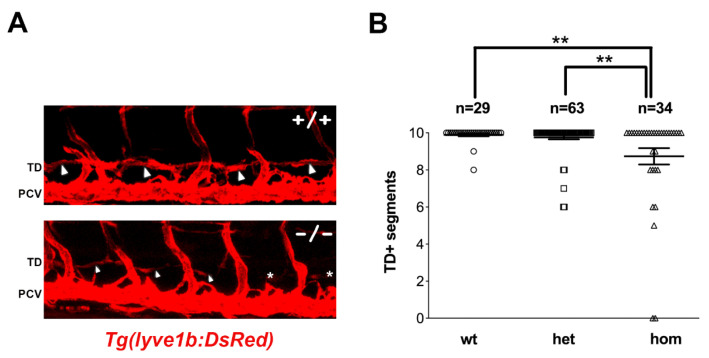
Homozygous *sox18* mutants show subtle but statistically significant defects in thoracic duct (TD) formation. (**A**) Confocal trunk images representing wt (+/+) and *sox18^sa12315^* homozygous mutant larvae (−/−) in the *Tg(lyve1b:DsRed)* line at 5dpf. Large and small white arrowheads point to TD+ segments (of typical or thinner aspect, respectively) while asterisks indicate the absence of TD. (**B**) The graph reports the mean number of TD+ segments, counted along 10 consecutive trunk segments, together with the Standard Error of the Mean (SEM), in all analyzed embryos of the three genotypes (wt: *sox18*^+/+^, het: *sox18*^+/−^, hom: *sox18*^−/−^). Data were gathered in three independent experiments, and each symbol represents the number of TD+ segments of a single analyzed larva. n = number of larvae, ** = *p* < 0.01. TD = thoracic duct; DA = dorsal aorta; PCV = posterior cardinal vein. Lateral view, anterior to the left.

**Figure 3:**
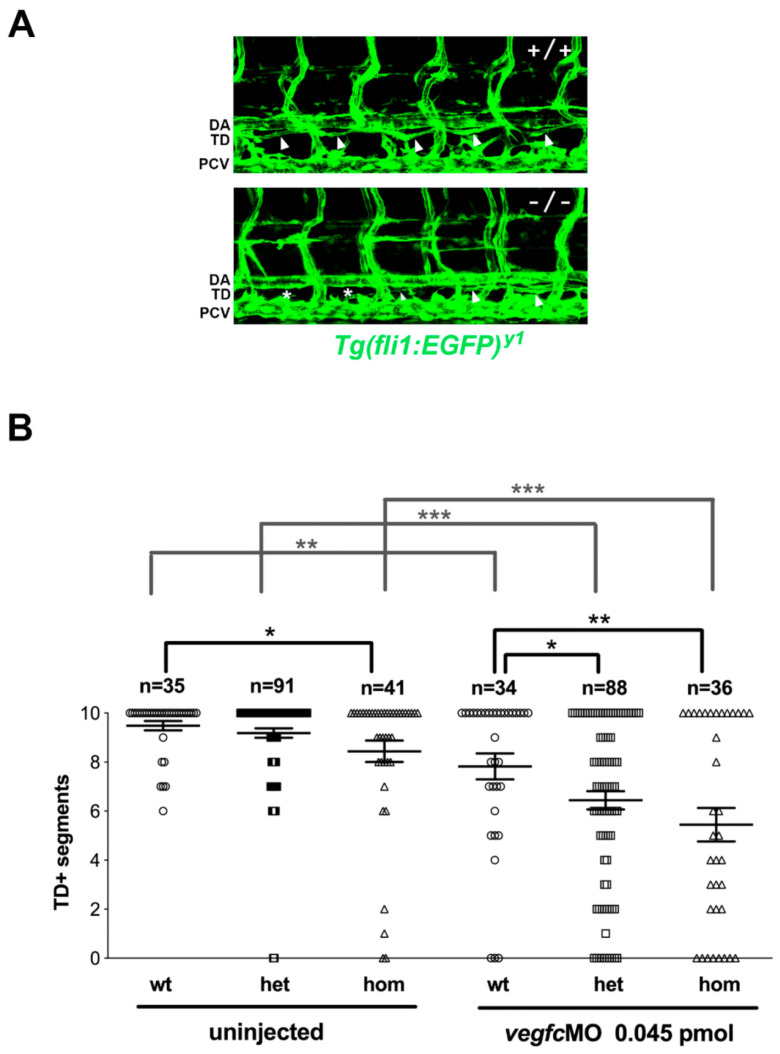
TD formation defects are exacerbated upon slight perturbation of Vegfc signaling. The progeny of *sox18^sa12315^* heterozygote matings in the *Tg(fli1a:EGFP)^y1^* line were injected with a subcritical dose of *vegfc*-MO or left uninjected; TD formation was analyzed at 5dpf. (**A**) Confocal trunk images of uninjected wt (+/+) and *sox18* homozygous mutant (−/−) larvae. Arrowheads point to TD+ segments, while asterisks indicate the absence of TD; a smaller arrowhead marks a thinner TD+ segment. (**B**) The graph reports the mean number of TD+ segments, counted along 10 consecutive trunk segments, together with the SEM, for all analyzed larvae of each genotype (wt: sox18^+/+^, het: sox18^+/−^, hom: sox18^−/−^). Each symbol represents the number of TD+ segments of a single larva; data were gathered in several independent experiments. Uninjected larvae on the left are compared to larvae with partially reduced Vegfc on the right. n = number of larvae, * = *p* < 0.05; ** = *p* < 0.01; *** = *p* < 0.001. TD = thoracic duct; DA = dorsal aorta; PCV = posterior cardinal vein. Lateral view, anterior to the left.

To address the impact of the *sox18^sa12315^* mutation on lymphatic development, we scored the formation of the thoracic duct (TD) at 5 dpf in the larvae derived from matings of heterozygous mutant fish. The *sox18^sa12315^* mutation was introduced in several transgenic reporter lines, i.e., *Tg(fli1:EGFP)^y1^*, *Tg(lyve1b:DsRed)^nz101^*, and *Tg(mrc1a:EGFP)^y251^;Tg(kdrl:mCherry)^y171^*, which allowed the visualization of blood and lymphatic vessels [18,39,40]. Lymphatic thoracic duct formation was scored in ten consecutive trunk segments of all larvae, which were subsequently genotyped, thus revealing a mild reduction in thoracic duct segments in homozygous *sox18^sa12315^* mutants compared to siblings (Figure 2 and Figure 3 uninjected, Figure S2A). Though modest, this reduction was sizeable and statistically significant in all reporter lines.

We previously reported that slight perturbations in VEGFC/VEGFR3 signaling, via partial knockdown of *vegfc* or *flt4* with morpholinos, synergize with the morpholino-mediated partial knockdown of *sox18* in inducing lymphatic defects in zebrafish [28] (Figure S1B). We therefore tested the impact of the microinjection of low doses of *vegfc*-MO in embryos derived from matings of heterozygous *sox18^sa12315^* mutant fish. Defects in the formation of the thoracic duct were exacerbated and became evident not only in homozygous but also in heterozygous *sox18^sa12315^* mutant larvae compared to wild-type larvae, using both the *fli1:EGFP* and the *lyve1b:DsRed* reporters (Figure 3B and Figure S3).

### 3.3. sox18^sa12315^ Mutants Have Milder Lymphatic Phenotypes Than sox18 Morphants

To help in the comparison of the effect of the *sa12315* mutation (this paper) with that of the previously reported *sox18*-MOs [28], we decided to plot the *sox18* morphant data in the *Tg(fli1:EGFP)* reporter line as they are now shown here for *sox18^sa12315^* mutants (compare Figure S1A with Figure 3). Moreover, we also directly compared thoracic duct formation in *sox18^sa12315^* mutants and in *sox18* morphants in the *Tg(mrc1a:EGFP);Tg(kdrl:mCherry)* double reporter line, in which both *sox18* mutation and knockdown had not been previously analyzed (Figure S2). Although statistically significant, the difference in the mean number of TD+ segments in *sox18* wild-type larvae versus homozygous mutant larvae is in the order of 10–20% in the two transgenic lines. On the other hand, there is an almost twofold to over threefold reduction in the mean number of TD+ segments in *sox18* morphants versus control larvae injected with a standard morpholino, depending on the transgenic reporter line (*Tg(fli1:EGFP)* and *Tg(mrc1a:EGFP);Tg(kdrl:mCherry)*, respectively).

Finally, given the great contribution of the maternal component in the phenotype of *prox1a* mutants [34], we decided to generate maternal-zygotic *sox18^sa12315^* mutants (hereafter MZ*sox18* mutants). Homozygous zygotic *sox18^sa12315^* mutants (Z*sox18*) are present at expected Mendelian ratios as young adults, in the progeny of *sox18^sa12315^* heterozygotes, and are fertile. MZ*sox18* mutants were therefore generated by crossing Z*sox18* mutant females and males and analyzed up to 5 dpf, to score for lymphatic thoracic duct formation. As shown in Figure S4A, there is a statistically significant reduction in the number of TD+ segments in MZ*sox18* mutants versus wild-type larvae. Nevertheless, TD formation defects appear comparable in MZ*sox18* mutants and in Z*sox18* mutants (compare Figure S4A and Figure 3).

Taken together, these data confirm that Sox18 plays a role in lymphatic development in zebrafish but highlight that *sox18^sa12315^* mutants have milder lymphatic phenotypes than *sox18* morphants.

### 3.4. Ectopic Expression of sox7 in the PCV of sox18^sa12315^ Mutants, but Not sox18 Morphants

**Figure 4:**
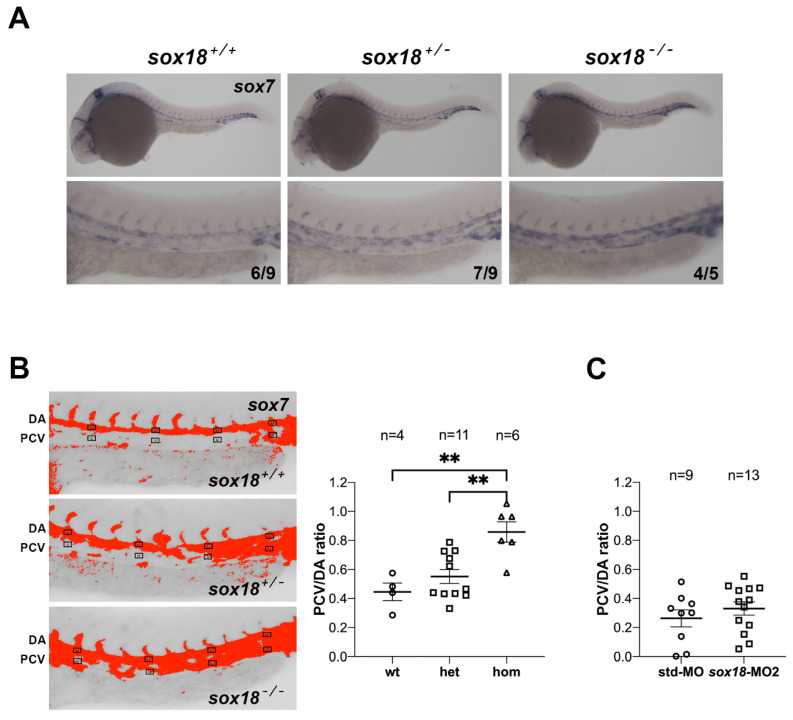
The expression of *sox7* in the PCV is upregulated in *sox18* mutants, but not in *sox18* morphants. (**A**) Representative images of *sox7* ISH on embryos at around 26 hpf derived from matings of *sox18^sa12315^* heterozygotes in the *Tg(lyve1b:DsRed)* line. Higher magnifications of the trunk region are shown below the full size images. Compared to wt embryos, the *sox7* ISH signal in the PCV is elevated in the great majority of *sox18*^−/−^ homozygotes and, to a lesser extent, in *sox18*^+/−^ heterozygotes. Numbers in each image state the number of embryos with the reported phenotype over the total analyzed embryos in one representative experiment. Lateral views, anterior to the left. Pictures were taken at 40× and 63× magnification, for lower and higher magnification images respectively. (**B**) Left, representative ImageJ-modified images, used to perform the quantification of the *sox7* ISH signal in the PCV and the DA (as described in Materials and Methods) on 26 hpf wt (+/+), *sox18^sa12315^* heterozygotes (+/−) or homozygous mutants (−/−). The graph on the right shows the calculated PCV/DA ratio in each embryo; embryos are grouped based on their genotypes: mean values and SEM are indicated. (**C**) The same analysis was performed on *sox7* ISH of *sox18* morphants and control embryos, as shown in Figure S6. The calculated PCV/DA ratio of each embryo is shown in the graph; mean values and SEM for std-MO injected embryos and *sox18* morphants are indicated. n = number of embryos, ** = *p* < 0.01; DA = dorsal aorta; PCV = posterior cardinal vein. The analysis was also repeated on ISHs of *sox18^sa12315^* mutants in the *Tg(fli1a:EGFP)^y1^* reporter line with similar results. ISH experiments on *sox18^sa12315^* mutants were repeated at least three times. Data shown in A and B were generated on different clutches of embryos.

When coexpressed, *sox18* and *sox7* play redundant roles in vascular development in zebrafish [29,30,31]. Moreover, in mice, *Sox7* and *Sox17*, which are not normally expressed during lymphatic development, can be activated in the absence of SOX18 function and act as modifiers of the lymphangiogenic defects caused by *Sox18* dysfunction in certain strains [15]. These data prompted us to analyze the expression of *sox7* in *sox18^sa12315^* mutants at developmental stages preceding lymphatic sprouting from the posterior cardinal vein (PCV). At these stages, in wild-type conditions, *sox7* ISH staining persists in the dorsal aorta (DA) and largely ends in the PCV (Figure 4A), as previously reported [28]. In homozygous zygotic *sox18^sa12315^* mutants, we noticed clearly elevated *sox7* staining in the PCV; moreover, most heterozygotes presented an intermediate *sox7* ISH signal in the PCV with respect to wild-type embryos and homozygous mutants (Figure 4A, Table S2). Quantification of *sox7* ISH signals in several small areas within the DA and the PCV to calculate PCV/DA ratios (Figure 4B) further highlighted the increased *sox7* expression in the PCV of zygotic *sox18^sa12315^* mutants. Remarkably, when the same analysis was performed on *sox7* ISHs of *sox18* morphants, no significant change was found with respect to controls injected with std-MO (Figure 4C and Figure S6).

We then checked for *sox7* expression in MZ*sox18* mutant embryos around 30 hpf. Here, again, the *sox7* ISH signal in the PCV is elevated in MZ*sox18* mutant embryos with respect to wild-type controls (Figure S4B).

We did not notice changes in *sox18* expression by ISHs between zygotic *sox18^sa12315^* mutants and wild-type siblings (Figure S5A): strong expression of *sox18* both in the DA and in the PCV is confirmed by PCV/DA ratio analysis. Similarly, endothelial *sox17* expression, which is confined to areas of the DA, is not elevated in the PCV of *sox18^sa12315^* mutants (Figure S5B).

We therefore conclude that the elevated *sox7* expression in the PCV of zygotic and maternal zygotic *sox18^sa12315^* mutants, but not *sox18* morphants, could partially compensate for the lack of functional Sox18 and explain the mild lymphatic phenotype of *sox18^sa12315^* mutants. We currently have no molecular explanation for the specific upregulation of *sox7* expression in the PCV of zebrafish *sox18* mutants. Since our ISH data do not reveal reduced *sox18* expression in zygotic *sox18* mutants, we tend to exclude the transcriptional compensation mechanism elicited by nonsense-mediated RNA decay [43,44]. Remarkably, *Sox7* expression is also upregulated in *Sox18* mutants in mice, in a strain-specific way [15], possibly pointing to an evolutionarily conserved mechanism to be further addressed.

## 4. Discussion

Knockdown studies and reverse genetic analyses have so far stimulated controversial conclusions on the role of Sox18 in early phases of lymphatic development in zebrafish [28,33]. Here, we address this issue using an independent loss-of-function allele, *sox18^sa12315^*. Our current data support the notion that Sox18 is indeed involved in lymphatic development in zebrafish, with a close interplay with the key Vegfc signaling, as already pointed out in knockdown studies and using a dominant-negative Sox18-*RaOp* mutant protein [28]. Thoracic duct formation defects, though statistically significant, are much milder in both zygotic and maternal-zygotic *sox18^sa12315^* mutants than in *sox18* morphants. Remarkably, in zygotic and maternal-zygotic *sox18^sa12315^* mutants, we found elevated expression of the closely related *sox7* gene in the PCV, with respect to wild-type embryos, at developmental stages preceding lymphatic sprouting. On the contrary, *sox7* expression in the PCV was unchanged in *sox18* morphants with respect to control embryos, and much lower in the PCV than in the DA. Apparently, *sox18* is the main *soxF* gene expressed in the PCV at developmental stages preceding lymphatic sprouting in wild-type conditions, but elevated expression of *sox7* can partially mask the lack of a functional Sox18 protein in *sox18* mutants, thus resulting in only mild lymphatic defects.

van Impel and colleagues [33] previously analyzed another *sox18* mutant allele, *sox18^hu10320^*, and reported no vascular or lymphatic defects at 5 dpf when scoring for thoracic duct formation. The *hu10320* mutation is a frameshift mutation upstream of the DNA-binding HMG-box domain coding sequence and is thus an LOF mutation, similarly to the *sa12315* mutation we characterize here, which introduces a stop codon within the HMG-box domain coding sequence and is thus predicted to give rise to a non-functional Sox18 protein. Since the authors did not report data on the expression of the other *soxF* genes in the *sox18^hu10320^* mutants, it is intriguing to speculate that *sox7* expression might also be elevated in that context, but this remains to be analyzed.

Even the presence of a single mutant *sox18^hu10320^* allele dramatically enhances the penetrance of the vascular circulatory phenotype in *sox7^hu5626^* homozygous mutants [45], and only *sox7*/*sox18* double homozygous mutants fully recapitulate the circulatory phenotype originally described in *sox7*/*sox18* double knocked-down embryos, where arterio-venous shunts result from incomplete acquisition of arterio-venous identity in endothelial cells [29,30,31]. This strengthens the notion that *soxF* genes can play redundant roles when coexpressed.

While our current work confirms a role for Sox18 in early trunk lymphatic development, a recent paper by Arnold and colleagues revealed that SoxF transcription factors are also involved in facial lymphatic development in zebrafish [46]. The authors show reduced facial lymphatic sprouting in *sox7*/*sox18* double partial morphants as well as in *sox7*/*sox18* double homozygous mutants (*sox7^hu5626/hu5626^*; *sox18^hu10320/hu10320^*) or even in *sox7* homozygous mutants lacking just one functional allele of *sox18* (*sox7^hu5626/hu5626^*; *sox18 ^+/hu10320^*).

The fine relative expression of *soxF* genes in different vascular beds, as well as their differential expression in mutant backgrounds, could therefore account for the relevance of each member of the F group of transcription factors in lymphatic development, as already shown in mice. Mice lacking SOX18 have an overt lymphatic phenotype in a pure C57BL/6 genetic background but are compensated by the strain-specific upregulation of *Sox7* and *Sox17* in the cardinal vein in a mixed 129-CD1 genetic background [11,15] Here, we report the elevated expression of *sox7* in the PCV in zebrafish *sox18^sa12315^* mutants, which show a milder trunk lymphatic phenotype than *sox18* morphants, while the expression of *sox17* appears to be unaffected. Zebrafish Sox17 is unique among SoxF proteins as it lacks the β-catenin interaction motif, which is otherwise present in all SOX group-F proteins [2,47]. Moreover, the temporal expression profile of *sox17* in embryonic and early larval stages diverges from that of *sox7* and *sox18* (Figure S2 in [29]), and its spatial expression in the vascular system appears to be much more marginal and restricted to the DA, while excluded from the PCV, at embryonic stages. Recently, a new role emerged for *sox17* in a novel vascularization process wherein blood vascular vessels arise from existing lymphatics in the anal fin, an adult-specific structure that is established at metamorphosis [48]. Das and colleagues report that mosaic overexpression of *sox17* in ECs negatively affected TD formation in injected embryos, pointing to a new role of Sox17 in the suppression of LEC fate.

The emerging picture is that SoxF proteins are collectively at play in processes involving EC plasticity (e.g., acquisition of arterial–venous identity, BEC to LEC transition, LEC to BEC transdifferentiation) and EC migration, but their relative contribution to these processes might be different in different organisms.

Nicenboim and colleagues [49] described a niche of specialized angioblasts in the PCV of zebrafish embryos, which gives rise to cells with lymphatic fates when induced by endodermal Wnt5b. When *wnt5b* was knocked down, the expression of *sox18*, *lyve1b* and *coup-tfII/nr2f2* was reduced in the PCV, together with the number of *prox1a*+ cells, whereas the expression of *vegfc* and *ccbe1* remained unchanged [49]. Interestingly, *wnt5b* morphants showed reduced PL+ and TD+ segments, but no alteration in venous ISVs [49]. This highlights a specific defect in lymphatic sprouting in *wnt5b* morphants, resembling what we described in *sox18* morphants [28].

Our previous knockdown studies suggested a genetic interaction between Sox18 and Vegfc to regulate lymphangiogenesis in zebrafish [28]. Combined partial knockdown of *sox18* and *vegfc*, using subcritical amounts of morpholinos, synergistically impaired venous and lymphatic sprouting from the PCV and parachordal lymphatic precursors at the horizontal myoseptum and interfered with TD formation. Although *sox18^sa12315^* mutants only show mild TD formation defects, the partial knockdown of *vegfc* greatly enhances the trunk lymphatic phenotype in *sox18^sa12315^* homozygous mutants but also reveals a statistically significant reduction in TD formation in *sox18^sa12315^* heterozygotes compared to wild-type siblings. This genotype-dependent effect of the partial knockdown of *vegfc* further strengthens the *sox18*–*vegfc* genetic interaction while confirming a role for Sox18 in zebrafish lymphatic development. The molecular mechanisms underlying this genetic interaction remain to be elucidated and warrant further studies.

SOX18 in mammals activates the expression of *Prox1* in a subset of cells of the CV, and when ectopically expressed in the CV, SOX7 and SOX17 are also able to induce Prox1 [11,15]. Not only is VEGFC signaling crucial for the budding and migration of Prox1+ lymphatic cells but VEGFC-mediated activation of VEGFR3 signaling is also necessary to maintain *Prox1* expression in LEC progenitors, and the *Prox1*-*Vegfr3* feedback loop controls the number of LEC progenitors and budding LECs [50]. In zebrafish, Vegfc plays a prominent role in regulating the division of bipotential Prox1+ precursor cells within the CV and upregulating Prox1 expression in daughter cells fated to become LECs [34]. When overexpressed, Vegfc is sufficient to induce *prox1a* expression in venous ECs, even if *sox18* and *sox7* are partially knocked down [34].

Our current data point to a more relevant role of Sox18 in zebrafish lymphatic development under limiting Vegfc conditions. It would be interesting to address whether Sox18 acts on Prox1 expression in the CV in zebrafish under slightly perturbed Vegfc conditions.

We previously reported that the heat-shock inducible expression of the dominant-negative SOX18–*RaOp* mutant protein in zebrafish causes impairment of lymphatic precursor sprouting from the vein and venous intersomitic vessel (vISV) formation, when induced at 29 hpf, just prior to secondary angiogenesis [28]. In heat-shocked *Sox18-RaOp* transgenic animals, we previously noticed a reduced level of *vegfc* RNA using qRT-PCR. ISH analysis of *vegfc* expression in *sox18* morphants at one specific developmental stage did not reveal variations with respect to control embryos in the DA region, but *vegfc* RNA injection could partially rescue the lymphatic phenotype of *sox18* morphants, thus pointing to a SoxF-VegfC axis [28].

Recently, SOX7 was shown to impact dermal lymphatic patterning in mice through direct and indirect regulation of *Vegfc* expression in blood ECs [51]. The authors point to fine-tuning of VEGFC levels produced in arteries, where SOX7 is expressed and can promote the expression of *Hey1*, a Notch effector that can repress the expression of *Vegfc*, or directly repress the expression of *Vegfc* through protein–protein interaction with HEY1 [51]. In their model, the loss of SOX7 function in arterial ECs was shown to cause an increase of endothelial VEGFC, which leads to more abundant and less polarized LEC progenitors in the CV, in the trunk, and to hyperproliferation of LEC progenitors and migration defects in dermal lymphatics.

The roles played by SoxF transcription factors in the regulation of Vegfc in zebrafish warrant future research to better elucidate evolutionarily conserved and non-conserved regulatory mechanisms.

## Data Availability

The authors declare that the data supporting the findings of this study are available within the article and its supplementary information file.

## Supplementary Materials

The following supporting information can be downloaded at: https://www.mdpi.com/article/10.3390/cells12182309/s1.
