# Supplementary material for: Lymphatic Defects in Zebrafish sox18 Mutants Are Exacerbated by Perturbed VEGFC Signaling, While Masked by Elevated sox7 Expression
Source: Cells. 2023 Sep 19;12(18):2309. doi: 10.3390/cells12182309 (PMC10527217; doi:10.3390/cells12182309)
Supplement: Supplementary file 1 [file cells-12-02309-s001.zip › cells-12-02309-s001.pdf]

Supplementary materials

Table S1. Partial knockdown of *sox7* causes a trunk-tail circulatory block in embryos carrying *sox18*^{sa12315} alleles

genotype	*sox7*-MO	trunk-tail circulation at 3 dpf		
		normal	reduced	absent
sox18 $^{+/+}$	+	5/5	-	-
sox18 $^{+/sa12315}$	+	5/11	-	6/11
sox18$^{sa12315/sa12315}$	+	-	-	7/7
sox18 $^{+/+}$	-	3/3	-	-
sox18 $^{+/sa12315}$	-	4/4	-	-
sox18$^{sa12315/sa12315}$	-	3/3	-	-

The table shows the circulatory phenotype in the trunk/tail region scored at 3dpf in the progeny of *sox18* heterozygous matings, either uninjected (-) or injected (+) with a subcritical dose of *sox7*-MO (0.125pmol/e) at 1-2 cells stage. All embryos were genotyped and results are presented grouping embryos with the same genotype and treatment.

Genotyping was also performed on a subset of non-circulating embryos in the progeny of a *sox18* heterozygous mating injected with *sox7*-MO (0.125pmol/e) shown in Figure 1B. Out of 14 randomly selected non-circulating embryos, we found 4 *sox18* heterozygotes and 10 *sox18* homozygous mutants.

Table S2. *sox18* mutants show elevated *sox7* expression in the PCV

genotype	*sox7* ISH signal in the PCV		
	weak	intermediate	strong
sox18 $^{+/+}$	13/16	3/16	0/16
sox18 $^{+/sa12315}$	8/36	21/36	7/36
sox18 $^{sa12315/sa12315}$	0/12	2/12	10/12

The table summarizes results obtained in three independent *sox7* ISH experiments performed on embryos at around 26-29hpf from matings of sox18 heterozygotes in the *Tg(lyve1b:DsRed)* reporter line. After staining, embryos were distributed in three categories based on the intensity of the *sox7* signal in the PCV (indicated as weak, intermediate, strong) and then genotyped. Images from an additional experiment showing a similar trend are displayed in Figure 4A.

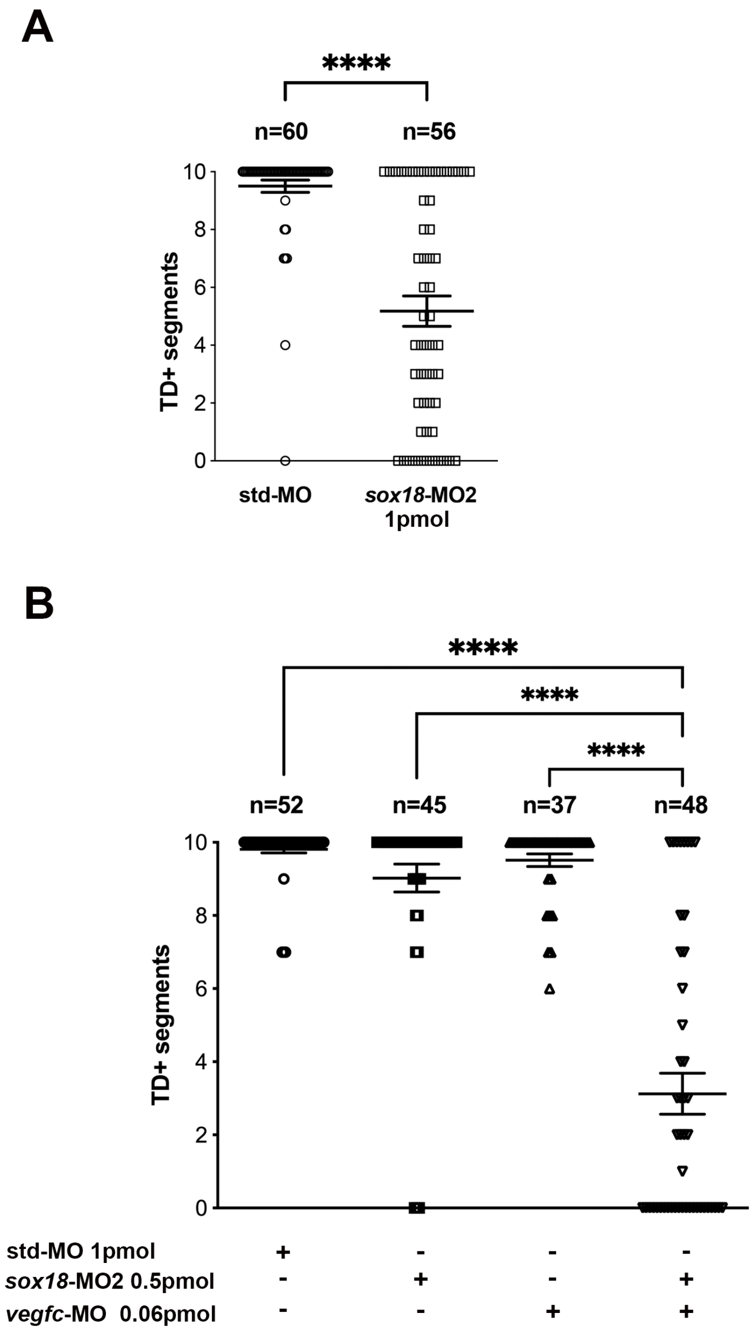

Supplementary Figure S1. New plotting of previously published data on *sox18* morphants to facilitate the comparison with new data on *sox18* mutants (this paper). A) TD+ segments of each *sox18* morphant and standard control embryo analyzed in Cermenati et al 2013 (Figure 1C in [28]) are plotted in the graph. **B)** TD+ segments data published in Cermenati et al 2013 (Figure 4B in [28]) and gathered injecting subcritical doses of *sox18* and *vegfc* MOs, either individually or in combination, are plotted showing mean values and SEM for each group of embryos.
n= number of larvae. ****= p<0.0001. In the graphs, mean values and SEM are indicated.

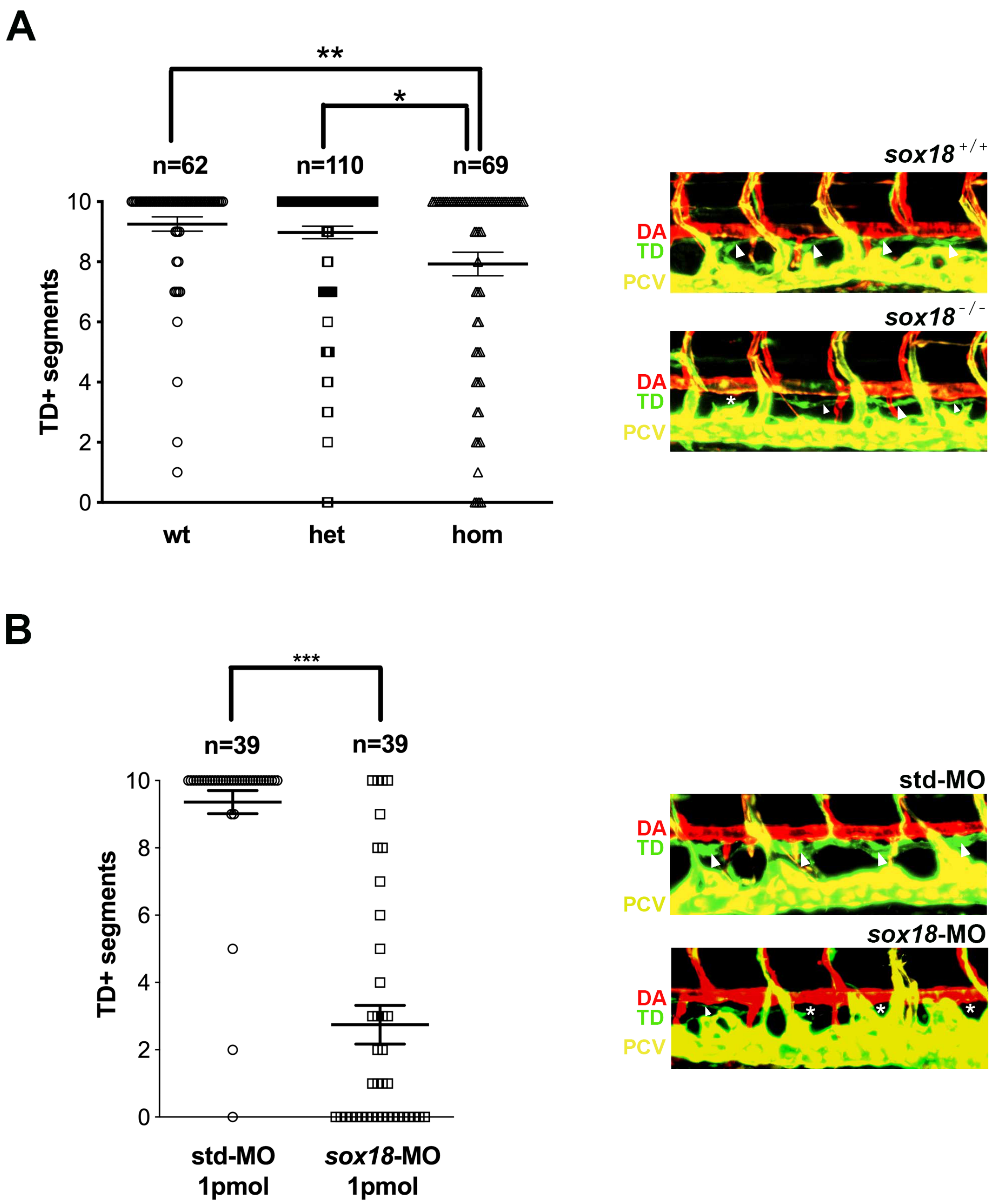

Supplementary Figure S2. *sox18* mutants and morphants present TD formation defects of different severity also in the *Tg(mrc1a:EFGP; kdrl:mCherry)* line. A) Homozygous *sox18* mutants (hom) show subtle but statistically significant defects in TD formation with respect to wt and heterozygous larvae (het). Three independent experiments were performed. On the right, representative confocal images of trunk regions of wt (*sox18*$^{+/+}$) and *sox18* homozygous mutant (*sox18*$^{-/-}$) larvae at 5dpf. **B)** The injection of *sox18*-MO2 causes strong TD formation defects in this transgenic reporter line. Data were obtained in two independent experiments. On the right, representative confocal images of a standard control larva and a *sox18* morphant at 5dpf. Lateral view, anterior to the left.

n= number of larvae. *= p<0.05; **= p<0.01; ***= p<0.001. TD= Thoracic Duct; DA=Dorsal Aorta; PCV= Posterior Cardinal Vein. Arrowheads point to TD+ segments, while asterisks indicate the absence of TD; smaller arrowheads mark thinner TD+ segments. In the graphs, mean values and SEM are indicated.

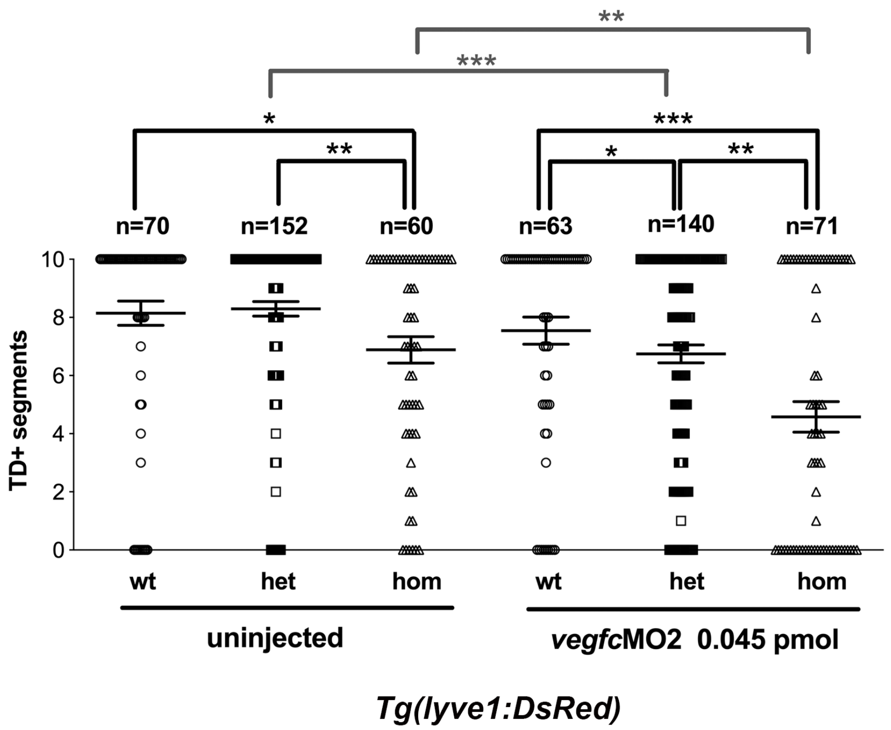

Supplementary Figure S3. When VegfC signalling is slightly perturbed, TD formation defects are exacerbated and become significant also in *sox18* heterozygotes even in the *Tg(lyve1b:DsRed)* reporter line. Data collected in three different experiments are plotted, indicating the number of TD+ segments for each analyzed larva in the progeny of *sox18* heterozygous matings, either uninjected or injected with subcritical doses of *vegfc*-MO at 1-2 cells stage. Mean values and SEM are shown.

n= number of larvae. *= $p<0.05$; **= $p<0.01$; ***= $p<0.001$.

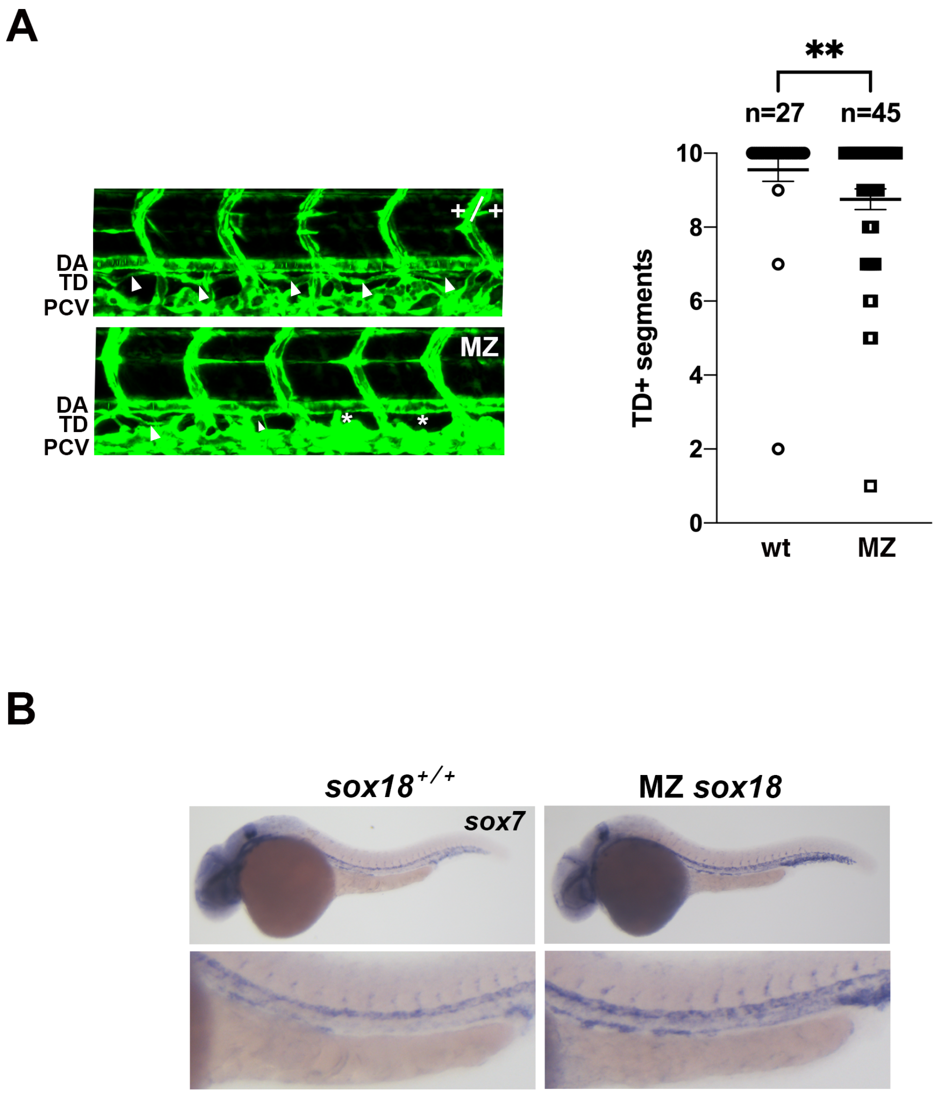

Supplementary Figure S4. Maternal-zygotic (MZ) *sox18* mutants show TD formation defects comparable to zygotic *sox18*^{sa12315} homozygous mutants. A) Representative confocal images of trunk regions of wt and MZ*sox18* mutant larvae. In the graph on the right, data obtained in four independent experiments are reported, plotting the number of TD+ segments for each analyzed larva. Mean values and SEM are shown for larvae of the indicated genotype. Arrowheads point to TD+ segments, while asterisks indicate the absence of TD; a smaller arrowhead marks a thinner TD+ segment. n= number of larvae. **= $p<0.01$. **B)** Representative ISH images and their magnification of the trunk region of 30hpf embryos, showing that *sox7* expression in the PCV is elevated in MZ*sox18* (right) with respect of wt control embryos (left). In this experiment, 9 out of 12 MZsox18 mutant embryos showed strong *sox7* staining in the PCV, while 7 out of 8 wt embryos showed low (4) or intermediate (3) *sox7* staining in the PCV. The ISH experiment was repeated three times on independent batches of embryos, with similar results. Lateral views, anterior to the left.

A

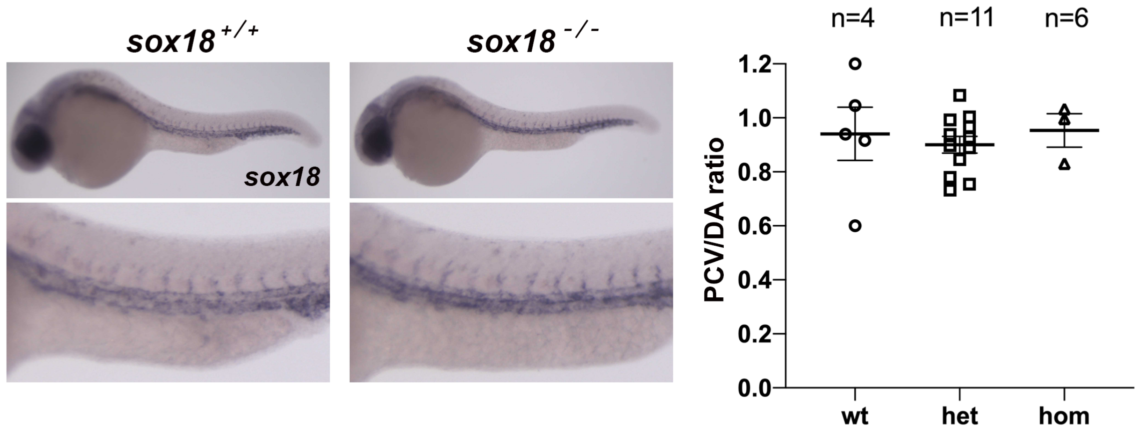

B

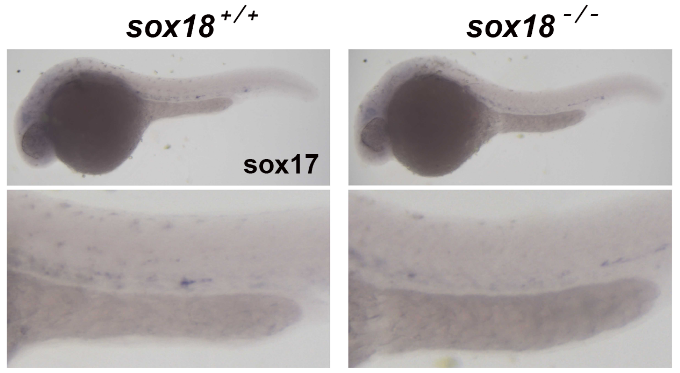

Supplementary Figure S5. The expression of *sox18* and *sox17* is largely unaffected in *sox18* mutants. **A)** Representative images of *sox18* ISHs on the progeny of *sox18* heterozygotes matings, with higher magnifications of the trunk region: comparable staining of DA, PCV and ISVs in wt (+/+) and *sox18* homozygous mutant (-/-) embryos at around 26hpf are shown on the left. On the right, PCV/DA ratios calculated for each embryo of this ISH experiment are plotted: mean value and SEM for *sox18* wt (+/+), het (+/-) and hom (-/-) embryos are shown. The *sox18* ISHs were repeated several times with similar results. **B)** Representative images of *sox17* ISHs on the progeny of *sox18* heterozygotes matings, with higher magnifications of the trunk region: a faint staining in the DA and no staining in the PCV is shown in both wt (+/+) and *sox18* homozygous mutant (-/-) embryos at around 26hpf. These ISHs were repeated twice. Lateral views, anterior to the left.

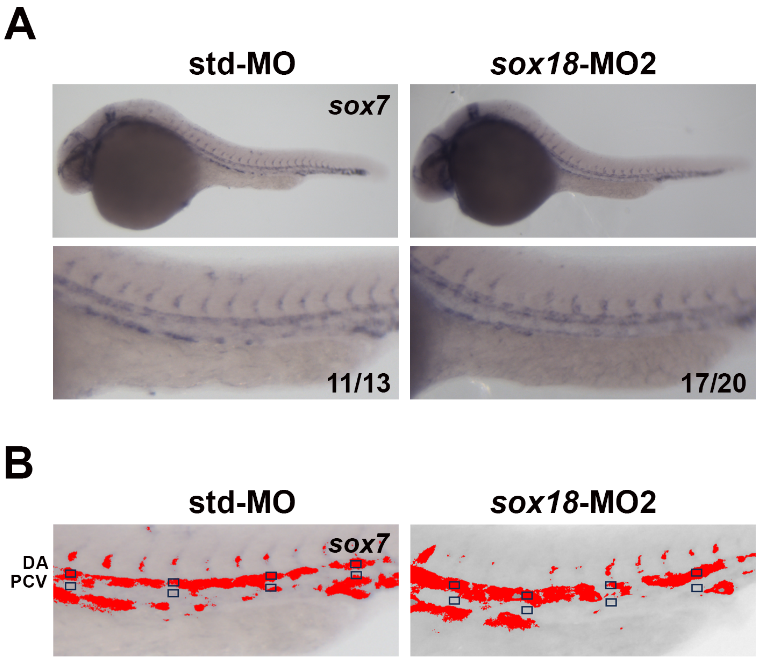

Supplementary Figure S6. The expression of *sox7* is largely unaffected in *sox18* morphants. **A)** Representative images of *sox7* ISHs on *sox18* morphants (right) and standard control embryos (left) at around 26hpf, with higher magnifications of the trunk region: comparable staining of DA, PCV and ISVs are shown. Numbers in each image state the number of embryos with the reported phenotype over the total analyzed embryos in one representative experiment. Several ISH experiments on *sox18* morphants and control embryos had been performed and previously published in Cermenati et al 2013 (Supplemental material Figure III in [28]). Lateral views, anterior to the left. **B)** Representative ImageJ-modified images, used to perform the quantification of the *sox7* ISH signal in the PCV and the DA (as described in M&M section) on *sox18* morphants (right) and standard control embryos (left) at around 26hpf. The corresponding graph, plotting PCV/DA ratios, is shown in Figure 4C.
